# PRSS3 is a prognostic marker in invasive ductal carcinoma of the breast

**DOI:** 10.18632/oncotarget.15590

**Published:** 2017-02-21

**Authors:** Li Qian, Xiangxiang Gao, Hua Huang, Shumin Lu, Yin Cai, Yu Hua, Yifei Liu, Jianguo Zhang

**Affiliations:** ^1^ Department of Clinical Pathology, Affiliated Hospital of Nantong University, Nantong, Jiangsu, China; ^2^ Department of Oncology, Affiliated Tumor Hospital of Nantong University, Nantong Tumor Hospital, Nantong, Jiangsu, China; ^3^ Research Center of Clinical Medicine, Affiliated Hospital of Nantong University, Nantong, Jiangsu, China

**Keywords:** invasive ductal carcinoma, immunohistochemistry, PRSS3, prognosis

## Abstract

**Objective:**

Serine protease 3 (PRSS3) is an isoform of trypsinogen, and plays an important role in the development of many malignancies. The objective of this study was to determine PRSS3 mRNA and protein expression levels in invasive ductal carcinoma of the breast and normal surrounding tissue samples.

**Results:**

Both PRSS3 mRNA and protein levels were significantly higher in invasive ductal carcinoma of the breast tissues than in normal or benign tissues (all *P* < 0.05). High PRSS3 protein levels were associated with patients’ age, histological grade, Her-2 expression level, ki-67 expression, and the 5.0-year survival rate. These high protein levels are independent prognostic markers in invasive ductal carcinoma of the breast.

**Materials and Methods:**

We used real-time quantitative polymerase chain reactions (*N* = 40) and tissue microarray immunohistochemistry analysis (*N* = 286) to determine PRSS3 mRNA and protein expression, respectively. PRSS3 protein levels in invasive ductal carcinoma of the breast tissues were correlated with the clinical characteristics of patients with invasive ductal carcinoma of the breast and their 5.0-year survival rate.

**Conclusions:**

PRSS3 acts as an oncogene in invasive ductal carcinoma of the breast development and progression. This finding implies that detection of PRSS3 expression can be a useful prognosis marker and the targeting of PRSS3 can potentially represent a new strategy for invasive ductal carcinoma of the breast treatment.

## INTRODUCTION

Breast cancer is one of the most common malignancies that threatens women's health and is now the number one malignant tumor among women. Studies have shown that [1] malignant tumors are caused by a variety of genes that are involved in multistage synergistic results. Over the past two decades, several studies have been conducted on the early diagnosis of breast cancer. Our study was focused on the tertiary prevention of cancer to determine the best adjuvant therapy to prevent post-surgery recurrence and metastasis [2–3]. Multidisciplinary scientists have collaborated to promote breast cancer treatment and have made great progress in mortality reduction with the use of endocrine therapy. However, further studies are needed to identify effective biomarkers, particularly among patients (estimated to account for 30-40% of general breast cancer population) who are insensitive to endocrine therapy [3–5]. Therefore, identifying useful biomarkers is essential for precision treatment of breast cancer.

The serine protease super family of chymotrypsin is expressed in intestinal digestion of food proteins in mammals [6]. These key enzymes are involved in digestion, fibrinolysis, reproduction, blood clotting, immune response, and signal transduction from the extracellular environment to the cell; however, there are many characteristics and functions that are not yet clear.

Serine proteases share a common chemical mechanism of having the same reactive serine residue catalytic site, but they differ greatly in degrees of native properties and substrate selectivity [7]. Chemokines, growth factors, growth factor receptors, and other signaling receptors are activated and transmitted through an extensive array of membranes and secreted serine proteases, and then enhance the transduction of signals for tumorigenesis, tumor proliferation, and metastasis [8–10].

Serine protease 3 (PRSS3) is one of the isoforms of trypsinogen, which plays an important role in the development and progression of many types of malignant tumors [17]. In 2010, Hockla et al. [7]. created an HMT-3522 breast cancer–growth model under 3-dimentional organ culture conditions and found that the expression of PRSS3 in breast cancer cell line T4-2 was enhanced. In our study, real-time quantitative polymerase chain reaction (qPCR) and tissue microarray immunohistochemistry analysis (TMA-IHC) were used to determine the mRNA and PRSS3 expression, respectively, in invasive ductal carcinoma of the breast from tissue samples. We sought to determine both the mRNA and protein expression of PRSS3 in invasive ductal carcinoma of the breast (IDC) tissue samples and correlate this with the patient's clinical characteristics and 5.0-year survival rate.

## RESULTS

### PRSS3 mRNA level was significantly higher in IDC tissues than in adjacent normal tissues

**Figure 1:**
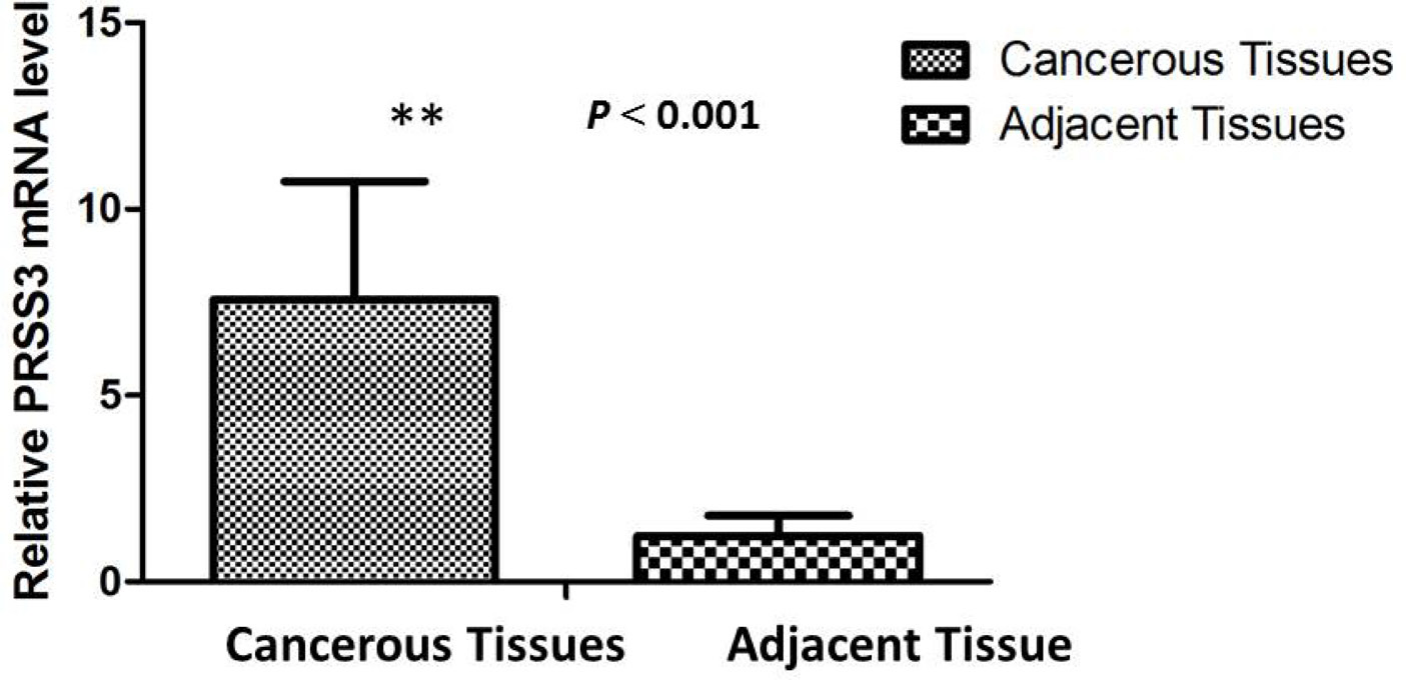
The relative mRNA expression of PRSS3 was significantly up-regulated in IDC tissues (*n* = 40) compared to matched adjacent non-cancerous tissues (*n* = 40) assessed by qPCR (*P* < 0.001) .

To determine the PRSS3 mRNA level in IDC patients, total RNA was isolated from the cancerous tissue of 40 IDC patients. Concurrently, 40 tissue samples of matched adjacent normal breast were collected to determine mRNA levels in noncancerous tissues. Real-time qPCR revealed that PRSS3 mRNA expression was upregulated in IDC samples compared with that in corresponding adjacent normal breast tissue samples. The expression of PRSS3 mRNA in IDC tissue samples (7.04 ± 2.04) was significantly higher than that in noncancerous tissues (2.39 ± 0.50) (*P* < 0.001) (Figure 1).

### PRSS3 expression was significantly higher in IDC than surrounding normal and benign tissues

**Figure 2:**
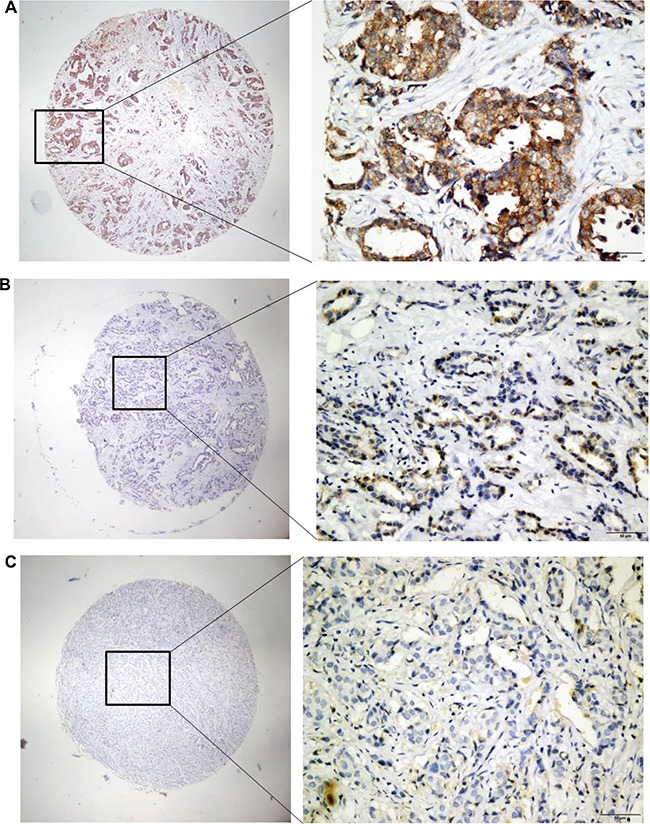
Analysis of PRSS3 expression and its cellular distribution by immunohistochemistry The expression of PRSS3 was analyzed on tissue microarrays by immunohistochemistry with the primary rabbit anti-human PRSS3 antibody. The positive PRSS3 expression with brown staining particles was distributed in the cytosol and cell nuclei, a1-a2) Strong straining of PRSS3 in IDC tissues; b1-b2) Mild straining of PRSS3 in IDC tissues; c1-c2) Light and Negative straining of PRSS3 in IDC tissues. a1, b1 and c1 are ×40 magnification (bar = 500 μm), a2, b2 and c2 are ×400 magnification (bar = 500 μm).

The overexpression of PRSS3 and its cellular distribution in IDC or surrounding tissues were analyzed by TMA-IHC using rabbit polyclonal antihuman PRSS3 antibodies (Figure 2). We determined PRSS3 protein expression in 386 archived breast tissue blocks comprising 286 IDC tissues and 100 normal breast tissues. Positive PRSS3 staining, identified as brown particles, was distributed in the cytosol and cell nuclei of the tissue samples. The incidence of PRSS3 expression in IDC tissue (66.43%, 190 of 286) was significantly higher than that in normal breast tissue (22%, 22 of 100) (χ2 = 14.773, *P* < 0.001). The frequency of positive PRSS3 expression in IDC tissue and in normal tissue is shown in Table 1.

**Table 1:** Incidence of PRSS3 expression in IDC tissues and normal breast tissues

Tissue sample	Case No.	PRSS3 expression level		*P* value
		Negative (*n*, %)	Positive (*n*, %)	
Normal breast tissue	100	78 (78.00)	22 (22.00)	< 0.001*
IDC	286	96 (33.57)	190 (66.43)	

### Association of PRSS3 expression with IDC clinical characteristics

We next investigated the relationship between PRSS3 protein levels and the clinical pathologic parameters of the patient (Table 2).

**Table 2:** Clinicopathological characteristics of high PRSS3 expression in 286 IDC patients

Groups	Patients, *n*	% of total	PRSS3 high expression, *n*	% of total	χ2	*P* value
Age (yr)					8.407	0.015
< 55	154	53.85	62	40.26		
≥ 55	132	46.15	34	25.76		
Pathology typing					15.718	0.003
I	36	12.59	12	33.33		
II	150	52.45	44	29.33		
III	100	34.97	40	40.00		
Tumor size (cm)					4.608	0.100
< 2	66	23.08	16	24.24		
≥ 2	220	76.92	80	36.36		
Axillary lymph node					0.637	0.727
N0	180	62.94	60	33.33		
NX	106	37.06	36	33.96		
ER expression					1.496	0.473
Negative	118	41.26	42	35.59		
Positive	168	58.74	54	32.14		
PR expression					3.708	0.157
Negative	124	43.36	42	33.87		
Positive	162	56.64	54	33.33		
Her-2 expression					7.899	0.019
Negative	110	38.46	36	32.73		
Positive	176	61.54	60	34.09		
Ki-67 expression					6.122	0.047
Low	112	39.16	47	41.96		
High	174	60.84	49	28.16		
Molecular typing					9.679	0.139
Luminal A	95	33.22	38	40.00		
Luminal B	105	36.71	32	30.48		
Her-2 positive	46	16.08	10	21.74		
Basal-like	40	13.99	16	40.00		
Five-year survival					11.165	0.004
Yes	238	83.22	70	29.41		
No	48	16.78	26	54.17		

High PRSS3 protein expression was significantly associated with patients’ age (*P* = 0.015), pathology typing (*P* = 0.003), Her-2 expression (*P* = 0.019), Ki-67 expression (*P* = 0.047), and the 5.0-year survival rate (*P* = 0.004).

Among the 286 IDC patients, we got 26 cases of triple-negative breast cancer (TNBC) patients; we correlated PRSS3 protein expression with TNBC patents’ clinical characteristics (Table 3). There were no significant associations between high PRSS3 protein expression and the clinicopathological features.

**Table 3:** Clinicopathological characteristics of high PRSS3 expression in 26 triple-negative breast cancer (TNBC) patients

Groups	Patients, *n*	% of total	PRSS3 high expression, *n*	% of total	χ2	*P* value
Age (yr)					0.650	0.723
< 55	18	69.23	6	33.33		
≥ 55	8	30.77	4	50.00		
Pathology typing					7.800	0.099
I	2	7.69	0	0.00		
II	6	23.08	4	66.67		
III	18	69.23	6	33.33		
Axillary lymph node					5.720	0.057
N0	20	76.92	8	40.00		
NX	6	23.08	2	33.33		
Ki-67 expression					4.030	0.133
Low	6	23.08	4	66.67		
High	20	76.92	6	30.00		
Five-year survival					0.087	0.958
Yes	20	76.92	8	40.00		
No	6	23.08	2	33.33		

### High PRSS3 protein expression predicts poor 5-year survival in IDC patients

**Figure 3:**
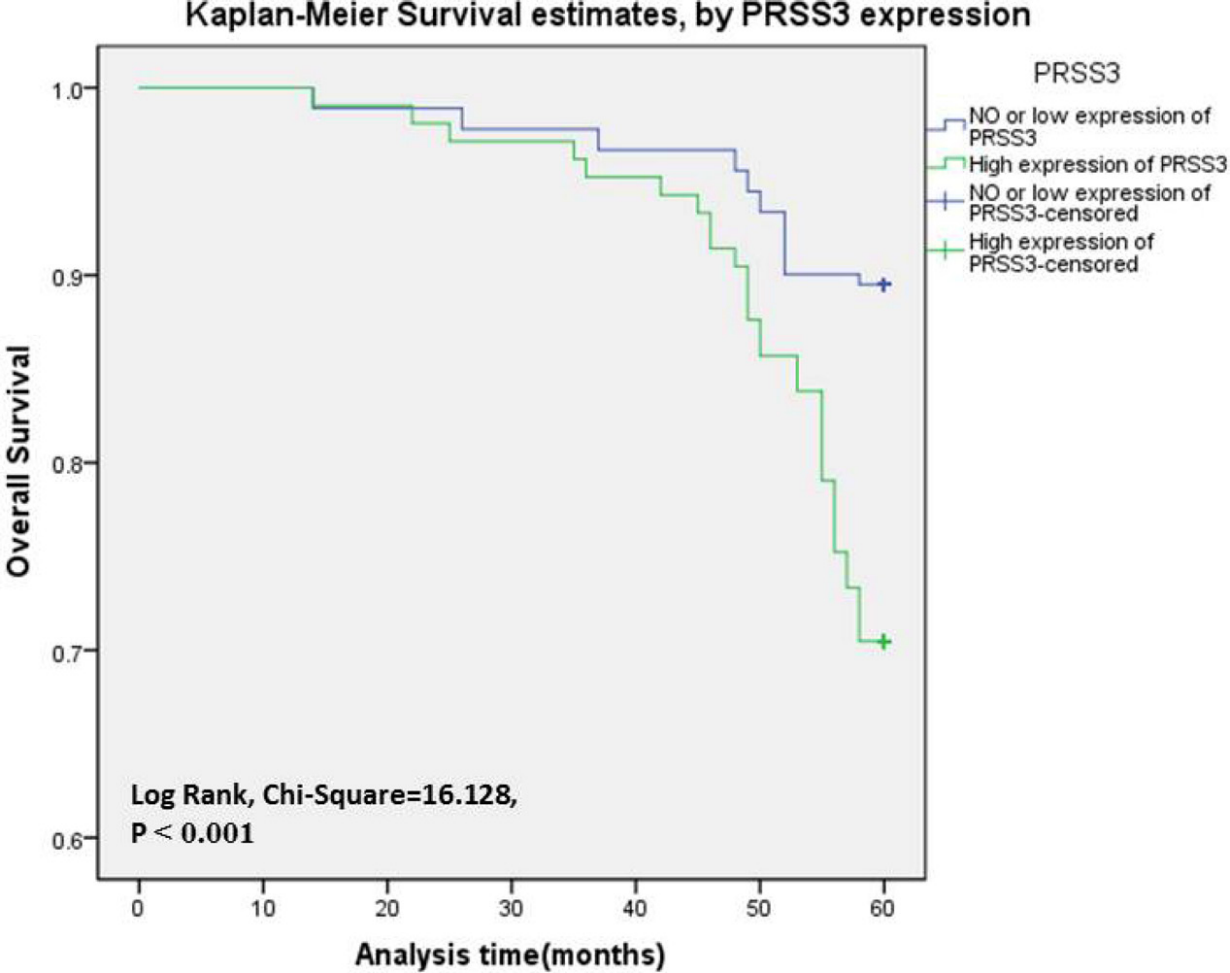
The PRSS3 expression curves were calculated by Kaplan-Meier method Survival curves of 286 IDC patients were made according to cancerous tissues expressing a low or high level of PRSS3 (log-rank test, *P* < 0.001). The green line is the high PRSS3 group; the blue line is the low and without PRSS3 group.

We analyzed the association between PRSS3 expression and the survival status of 286 patients using both univariate and multivariate analysis (Table 4). In univariate analysis, the overexpression of PRSS3 showed a significant relationship with a poor 5.0-year survival rate of 286 IDC patients (*P* = 0.007). Meanwhile, specific IDC clinical prognostic factors, such as Her-2 expression level (HR, 2.805, 95% confidence interval [CI]: 1.337–5.885; *P* = 0.006), ki-67 expression level (HR, 0.455, 95% CI: 0.240-0.859; P = 0.015), and molecular typing (HR, 1.725, 95% CI: 1.096–2.714; *P* = 0.019), also showed a statistically significant correlation with the 5.0-year survival rate based on the univariate Cox regression univariate analysis. All of these factors were enrolled in a multivariable analysis. Higher PRSS3 expression (HR, 0.372, 95% CI: 0.178–0.781; *P* = 0.009), Her-2 expression level (HR, 2.588, 95% CI: 1.277–5.246; *P* = 0.008), and ki-67 expression level (HR, 0.492, 95% CI: 0.269–0.901; *P* = 0.022) remained significantly associated with a poor 5.0-year survival rate, so they were classified as independent predictive factors for poor IDC outcome. The Kaplan-Meier Survival Curves demonstrated that IDC patients with high PRSS3 expression had a significantly poorer 5.0-year survival rate compared to those with low or no PRSS3 expression (Figure 3).

**Table 4:** Univariate and multivariable analysis of prognostic factors in IDC patients for 5-year survival

Variable	Univariate analysis			Multivariable analysis		
	HR	*P* > |z|	95% CI	HR	*P* > |z|	95% CI
Age (yr)						
< 55 vs. ≥ 55	1.371	0.320	0.736–2.553			
Pathology typing						
I vs. II –III	0.829	0.692	0.327–2.102			
Tumor size (cm)						
< 2 vs. ≥ 2	1.224	0.614	0.559–2.678			
Axillary lymph node						
N0 vs. NX	1.060	0.858	0.561–2.001			
ER expression						
Positive vs. Negative	1.480	0.448	0.538–4.069			
PR expression						
Positive vs. Negative	1.571	0.361	0.596–4.142			
Her-2 expression						
Positive vs. Negative	2.805	0.006	1.337–5.885	2.588	0.008	1.277–5.246
Ki-67 expression						
High vs. Low	0.455	0.015	0.240–0.859	0.492	0.022	0.269–0.901
Molecular typing						
Luminal A vs. Luminal B vs. Her-2 positive vs. Basal-like	1.725	0.019	1.096–2.714	1.314	0.052	0.998–1.730
PRSS3 expression						
High vs. Low	0.346	0.007	0.159–0.752	0.372	0.009	0.178–0.781

Besides, in univariate analysis, there were no significant correlations between the overexpression of PRSS3 and the 5-year survival of the 26 triple-negative breast cancer patients (*P* = 0.390).

## DISCUSSION

In the current study, we determined the levels of both PRSS3 mRNA and PRSS3 protein expression in both IDC tissues and adjacent normal tissues. The PRSS3 mRNA levels were significantly higher in IDC tissues than in adjacent tissues. Similarly, the PRSS3 protein levels were significantly higher in IDC tissues than in adjacent normal tissues. High PRSS3 protein levels were associated with patients’ age, Her-2 expression levels, ki-67 expression levels, and pathology typing. Finally, high PRSS3 protein expression is an independent prognostic marker for a poor 5.0-year survival rate in IDC patients.

PRSS3, one of the three major isoforms of trypsinogen, is a serine protease that is synthesized mainly in pancreatic acinar cells. It can be secreted into the small intestine to promote digestion [15–16, 47]. As a member of the serine protease family, PRSS3 is highly homologous to trypsinogen I and II (PRSS1 and PRSS2) at both the gene and protein levels [17]; however, unlike PRSS1 and PRSS2, a small portion of pancreatic exocrine secretions is composed of PRSS3, which accounts for 3.0–10% of the trypsinogen content in normal pancreatic juice [18]. The tumor specificity of trypsinogen has been elucidated in many types of tumors and it is thought to be involved in the development and progression of malignancies [9, 11, 19–26]. PRSS3 is expressed in the airway epithelium and its active trypsin is detected in the lung bronchial epithelium, which might be related to its ability to activate various proteases in relation to coagulation, fibrinolysis, and/or inflammation [27]. In non-small–cell lung cancer (NSCLC), the expression of PRSS3 is closely associated with metastasis and with a low prognosis for NSCLC patients. In addition, overexpression of PRSS3 in lung-cancer tissue results in increased migration across endothelial cells, thereby highlighting the potential role of trypsin in tumor metastasis [28].

Promoter methylation of the PRSS3 gene suggests silencing of its expression, which is at ∼53% in NSCLC; however, some researchers believe that methylation in NSCLC is a random event [17]. While a study of esophageal squamous cell carcinoma found that promoter methylation can silence the expression of PRSS3, [29] some other studies reported that methylation of the PRSS3 promoter in bladder cancer, lung cancer, esophageal cancer, and gastric cancer also silence its expression [17, 29–30]. Some tissue microarray analyses have been conducted to confirm that PRSS3 is overexpressed in metastatic lung cancer [28]. Methylated PRSS3 might be a suitable gene for the detection of malignancies, although more studies are needed to elucidate the specificity of this gene for the detection of serum PRSS3 in cancer patients [17].

PRSS3 was expressed in both ovarian epithelial cancer and benign ovarian tumors, but its expression was significantly higher in ovarian epithelial cancer [31–32]. Hockla et al. [33–36]. demonstrated using nude mouse models that PRSS3 is highly expressed in prostate cancer and is a key factor in lung metastasis of prostate cancer. By reducing the expression of PRSS3, lung metastasis of prostate cancer would be significantly inhibited [35]. In addition, the researchers also found that PRSS3 is also expressed in the ovarian stroma, suggesting that the expression of PRSS3 might also be associated with the tumor's microenvironment [37–38]. In in vitro experiments using a cell model, Jiang et al. [36]. found that overexpression of PRSS3 increased the invasive ability of pancreatic cancer cells, but that when PRSS3 expression was knocked down, the invasion of pancreatic cancer cells significantly decreased; however, the expression of PRSS3 had no significant effect on the ability of cells to migrate. PRSS3 is expressed in the acinar cells of the pancreas but not in the normal ductal epithelium, and PRSS3 expression was significantly enhanced in cancer tissues with vascular and lymphatic metastasis. In pancreatic cancer, the research data showed that the expression of PRSS3 and lymph node metastasis, as well as distant metastasis, were closely related [36]. Our data suggest that PRSS3 acts as oncogenes in IDC.

Studies have shown that trypsin can activate protease-activated receptors in epithelial cells [39], thereby triggering the development of breast cancer through the signaling pathway [40–43] ; however, another study questioned this claim [44]. In colorectal cancer, trypsin suppresses the proliferation of cancer cells by blocking downstream gene proliferation-1 (BOP-1) [45]. Hockla et al. [7] found a series of trypsin-regulated genes using microarray analysis and real time q-PCR. Among them, EGFR, ITHAV, and CD109 were upregulated by trypsin expression, whereas BOP-1 and CD74 were downregulated. EGFR and its EGF-responsive family members were found to play a central role in the progression of breast and other tumors [46].

Our current study indicated that PRSS3 is an independent prognostic factor in the development and progression of IDC. In subsequent work, in vivo and vitro mechanisms will be necessary to further confirm and expand these conclusions. We look forward to a more comprehensive understanding of the development process of IDC so as to provide new findings that will help prevent and treat the disease.

### Study limitations

Our study had several limitations. First, our research samples were limited to the female Chinese population. We have not collected tissues from male patients, which might result in sample bias; therefore, our results cannot be extended to other countries or to males, and we do not have international research collaborations with larger and more diverse populations. Secondly, our study examined only IDC in breast cancer. As we know, a tumor is a heterogeneous disease and different types of tumors will have different prognoses; therefore, we do not know whether our findings can be generalized to all types of invasive breast cancer. Finally, we have not provided the mechanism by which PRSS3 occurs in IDC. In the next phase of the study, we intend to further validate the expression of PRSS3 using a cell model and a nude mouse model, which can aid in exploring its molecular mechanism of action in regulating the growth of IDC tumor cells.

## MATERIALS AND METHODS

### Human tissue specimens and patient clinical information

Human breast cancer and surrounding tissues in the study were obtained from 286 IDC patients, who underwent operations between January 2010 and August 2011 at the Affiliated Hospital of Nantong University, Jiangsu Province, China. The patient cohort inclusion and exclusion criteria included: (1) accurate pathologic diagnosis of primary IDC; (2) complete clinic pathologic and follow-up date; and (3) previously untreated, with surgery as the first treatment (Table 5). Thus, analysis of the data would reflect the actual impact of tumor biology on the clinical outcome. This study was approved by the Ethics Committee permission of the Affiliated Hospital of Nantong University, and all patients signed informed consent. The related important clinical information of each patient was collected from their medical records.

**Table 5:** Clinical characteristics of IDC patients

Parameter	*n*
Age (yr)	
Media (range)	55 (20–83)
Pathology typing	
I	36
II	150
III	100
Tumor size (cm)	
< 2	66
≥ 2	220
Axillary lymph node	
N0	180
NX	106
ER expression	
Negative	118
Positive	168
PR expression	
Negative	124
Positive	162
Her-2 expression	
Negative	110
Positive	176
Ki-67 expression	
Low	112
High	174
Molecular typing	
Luminal A	95
Luminal B	105
Her-2 positive	46
Basal-like	40
Five-year survival	
Yes	238
No	48

### PRSS3 expression and statistical analysis

PRSS3 mRNA level was determined by real-time quantitative PCR (qPCR). The primers sequences are as follows: PRSS3 forward primer (5′- TGCGCCATTGGTTTTCCATC-3′) and PRSS3 reverse primer (5′- ATACCACCCACTGTTCGCTG-3′), PRSS3 protein expression in tissue blocks was determined using tissue microarray immunohistochemistry (TMA IHC). Rabbit polyclonal anti-human PRSS3 antibody was used (dilution 1:50, ab170361, Abcam, USA). The PRSS3 protein level was quantified using a two-level grading system: the percentages for PRSS3 -positive cells were scored as follows: 0 (< 10%), 1 (10%–25%), 2 (26%–50%), and 3 (≥ 50%). Staining intensity was stratified into four categories: 0 (negative), 1 (weakly), 2 (moderate), and 3 (strongly). The sum of the percentage and intensity score was defined as the IHC staining score. According to above criterion, IDC tissues with PRSS3 expression were divided into two groups: 0–3 scores (low expression) and 4–9 scores (high expression). χ^2^ tests were performed to determine the correlation between PRSS3 expression and clinicopathologic parameters. Univariate and multivariate Cox regression models were used to identify prognostic factors. Kaplan-Meier method was used to calculate survival curves. For all analyses, a *P*-value < 0.05 was regarded as statistically significant. Data were analyzed using SPSS 19 statistics software (SPSS Inc., Chicago, IL, USA).
